# Imaging Diagnosis of a Lumbar Hernia: A Rare and Challenging Presentation

**DOI:** 10.7759/cureus.69130

**Published:** 2024-09-10

**Authors:** Arjun Aravindh Ramesh, Magesh Chandran, Madan Sundar, Evangeline P Christina, Karpagam Kannadasan

**Affiliations:** 1 Department of Surgery, Sree Balaji Medical College and Hospital, Chennai, IND; 2 Department of Radiology, Saveetha Medical College and Hospital, Saveetha Institute of Medical and Technical Sciences (SIMATS) Saveetha University, Chennai, IND

**Keywords:** perinephric fat herniation, posterior abdominal wall defect, prolene mesh repair, superior lumbar hernia, valsalva maneuver

## Abstract

Lumbar hernias, a rare form of abdominal wall hernia, typically present with subtle, gradually enlarging masses in the lumbar region, often overlooked due to their rarity. This case report details a 38-year-old male farmer who experienced a 10-month history of a slowly enlarging, non-tender swelling in the left loin area, which became more prominent during the Valsalva maneuver. Despite having no significant medical history or prior trauma, a CT scan revealed a 2 cm defect in the superior lumbar triangle, with herniation of perinephric fat, confirming the diagnosis of a superior lumbar hernia. Surgical intervention was undertaken, where a 3 × 3 cm defect was identified intraoperatively, matching the preoperative imaging findings. The hernia was repaired using a Prolene mesh, which was securely anchored to the surrounding posterior abdominal wall tissues to prevent recurrence. The patient’s postoperative recovery was smooth and without complications. This case underscores the necessity of including lumbar hernias in the differential diagnosis of lumbar masses, particularly in patients lacking common risk factors, and highlights the critical role of CT imaging in accurate diagnosis and surgical planning. Given the risks of incarceration or strangulation, early surgical repair with mesh reinforcement is essential for ensuring a successful outcome.

## Introduction

Lumbar hernias are an exceptionally rare type of abdominal wall hernia, accounting for fewer than 2% of all reported hernias. These hernias occur through defects in the lumbar region, either in the superior or inferior lumbar triangle. Inferior lumbar hernias are more common than superior lumbar hernias. The rarity of lumbar hernias, combined with their often subtle presentation, can make diagnosis challenging [1]. Unlike more common types of hernias, lumbar hernias do not typically present with pain, and their growth is usually slow and progressive, making them easy to overlook or misdiagnose, especially in patients without a history of trauma or significant medical conditions.

The complex anatomy of the lumbar region, including its muscular and fascial layers, contributes to the unique characteristics of lumbar hernias. These hernias can occur due to congenital weaknesses, post-surgical complications, or spontaneous occurrences [2]. They are most often seen in adults and can present with a wide range of symptoms depending on the size of the hernia and the structures involved. The superior lumbar triangle, bordered by the 12th rib, internal oblique muscle, and quadratus lumborum muscle, is a common site for these hernias, where they typically involve the herniation of fat or other abdominal contents; however, inferior lumbar hernias are more common than superior lumbar hernias.

In this case report, we describe a 38-year-old male farmer who presented with a progressively enlarging, non-tender swelling in the left loin over a 10-month period. The swelling, which became more prominent during the Valsalva maneuver, was diagnosed as a superior lumbar hernia on CT imaging. Given the potential complications associated with untreated lumbar hernias, such as incarceration or strangulation of the herniated contents, the patient underwent surgical repair using a Prolene mesh [3]. This case highlights the importance of considering lumbar hernias in the differential diagnosis of lumbar masses, especially in patients presenting with slow-growing, reducible swellings in the lumbar region.

## Case presentation

A 38-year-old male farmer presented to our district headquarters hospital with a complaint of a progressively enlarging swelling in the left loin over the past 10 months. The patient reported that the swelling had gradually increased in size but remained painless and reducible. There was no associated history of trauma, fever, weight loss, or any bowel or urinary complaints. The patient had no significant past medical or surgical history that could be related to his current condition.

**Figure 1:**
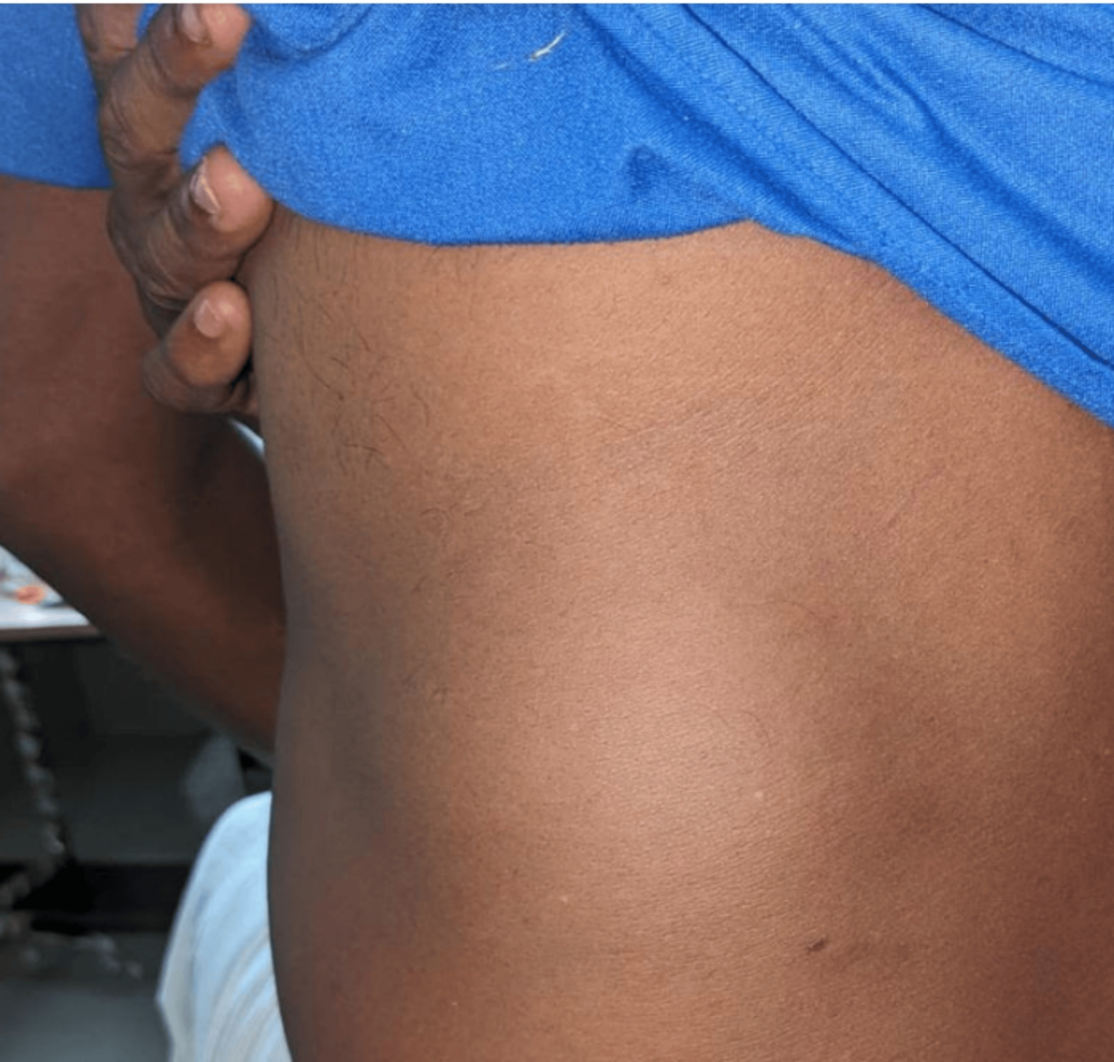
Swelling noted in left loin region.

On physical examination, the swelling was noted to be ovoid in shape, measuring approximately 5 × 5 cm. It was soft in consistency, non-pulsatile, non-tender, and reducible, and a positive cough impulse was present. The swelling was located in the superior lumbar triangle (Figure 1) and became more prominent during the Valsalva maneuver, a key finding that raised suspicion of a lumbar hernia. The rest of the physical examination was unremarkable, with no other abnormalities detected.

**Figure 2:**
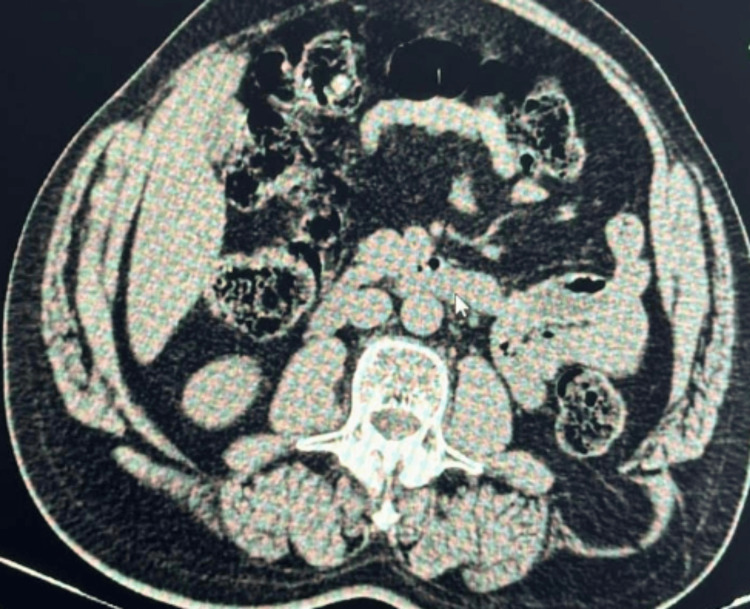
CT scan of the abdomen (axial section) reveals a defect in the left lumbar region within the superior lumbar triangle. This defect is associated with the herniation of perinephric fat through the posterior abdominal wall, confirming a lumbar hernia. A defect measuring approximately 2 cm in the left lumbar region with herniation of perinephric fat through the defect.

A CT scan of the abdomen and pelvis revealed a defect measuring approximately 2 cm in the left lumbar region, with herniation of perinephric fat through the defect (Figure 2). These findings were consistent with a diagnosis of a superior lumbar hernia.

**Figure 3:**
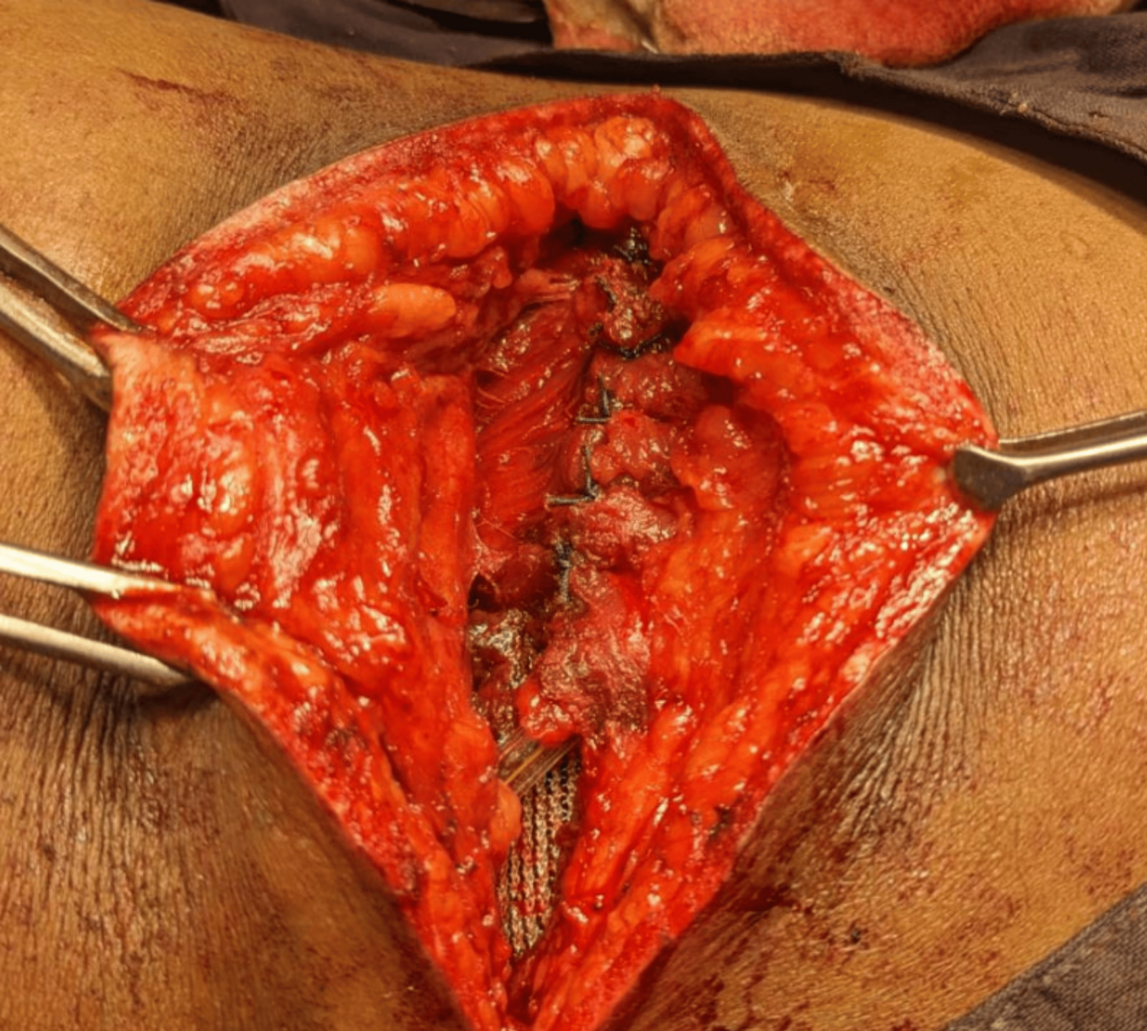
Intraoperative image.

During the surgery, the posterior abdominal wall was carefully opened in layers, revealing a defect measuring approximately 3 × 3 cm. The herniated perinephric fat was successfully reduced, and the defect was closed using a Prolene mesh, which was securely anchored to the posterior abdominal wall to reinforce the area and prevent recurrence (Figure 3).

Postoperatively, the patient’s recovery was uneventful. He was discharged with advice on wound care and was instructed to avoid heavy lifting and strenuous activities during the recovery period.

## Discussion

Lumbar hernias are an exceptionally rare subset of abdominal wall hernias, representing fewer than 2% of all hernia cases. Their infrequency, combined with a typically subtle and insidious onset, often leads to underdiagnosis, as the gradual progression of symptoms can easily be overlooked. These hernias arise from defects in the lumbar region, most commonly within the anatomical boundaries of the superior and inferior lumbar triangles [4]. An inferior lumbar hernia is a rare type of abdominal wall hernia that occurs in the lower back, typically through defects in the superior or inferior lumbar triangles. The inherent anatomical vulnerabilities in these regions make them susceptible to herniation, particularly in individuals exposed to activities that increase intra-abdominal pressure, such as heavy lifting or straining. The rarity and slow, often painless, development of lumbar hernias pose significant diagnostic challenges for clinicians, emphasizing the need for a high index of suspicion when evaluating patients with unexplained lumbar masses or discomfort. Accounting for fewer than 2% of all hernia cases, these hernias often present subtly with symptoms such as localized back pain, a palpable mass in the lumbar area, and discomfort radiating to the flank or lower abdomen, particularly exacerbated by activities that increase intra-abdominal pressure. Diagnosis can be challenging due to the gradual onset of symptoms, requiring imaging studies such as MRI or CT scans to identify the hernia and assess any associated nerve or tissue compression. Initial treatment typically involves conservative measures such as physical therapy and pain management, but surgery may be needed if symptoms persist or complications arise. Clinicians must remain vigilant for this rare condition when evaluating patients with unexplained lumbar discomfort or masses.

The differential diagnosis of swelling in this region includes lipoma, incisional hernia, and pseudo-hernia. When evaluating a swelling in the lumbar region, several conditions should be considered. Lipoma is a benign tumor composed of fatty tissue, presenting as a soft, movable mass under the skin that is typically non-tender and grows slowly. An incisional hernia occurs at the site of a previous surgical incision where abdominal contents protrude through the weakened area, often presenting as a bulge at the surgical scar, which may increase in size with straining or lifting. Pseudo-hernia, or a false hernia, is a condition where a bulge appears similar to a hernia but lacks a true hernial sac; it often results from muscular or fascial weakness without a protrusion of internal organs. Distinguishing these conditions involves careful physical examination and imaging studies to determine the precise nature of the swelling and guide appropriate treatment. The diagnosis of lumbar hernias typically necessitates advanced imaging studies, as physical examination alone frequently lacks the resolution required to identify these deep-seated defects. In the presented case, CT played an indispensable role in confirming the diagnosis. The CT imaging revealed a 2 cm defect in the superior lumbar triangle with herniation of perinephric fat, a finding consistent with other documented cases of lumbar hernia. CT imaging is regarded as the gold standard for diagnosing lumbar hernias due to its superior ability to delineate the hernia’s size, location, and nature of the herniated contents [5]. In this case, the identification of perinephric fat as the herniated material is significant, as it underscores the anatomical complexities involved and aids in preoperative planning. The precision with which CT imaging can map the herniated structures and define the anatomical defect is crucial for surgical decision-making, ensuring that the surgical approach is appropriately tailored to the patient’s specific condition.

Surgical intervention is the definitive treatment for lumbar hernias, primarily due to the risk of serious complications such as incarceration or strangulation of the herniated contents, which can lead to severe morbidity. When addressing hernias, including inferior lumbar hernias, surgeons may opt for either laparoscopic (transabdominal preperitoneal (TAPP)) or open surgical approaches based on the hernia’s specifics, patient health, and surgeon expertise. The laparoscopic TAPP technique involves making small incisions to insert trocars and a laparoscope, allowing repair of the hernia from within the abdominal cavity with minimal tissue disruption. This approach offers benefits such as reduced postoperative pain, quicker recovery, and smaller scars but requires advanced skills and provides a less comprehensive view compared to open surgery. Conversely, the open approach involves a larger incision to directly access and repair the hernia, offering excellent visibility and suitability for complex cases. While it provides a more straightforward view and is versatile for various hernia types, it results in a larger wound, a longer recovery period, and more postoperative pain. Both methods aim to effectively repair hernias, with the choice influenced by the hernia’s characteristics and the surgeon’s judgment. The surgical approach generally involves reducing the herniated contents back into the abdominal cavity and repairing the defect. In this case, the surgical team opted for a layered dissection of the posterior abdominal wall to expose the defect, which was ultimately found to measure approximately 3 × 3 cm, larger than initially estimated on imaging [6]. This discrepancy between preoperative imaging and intraoperative findings is not uncommon and highlights the need for flexibility in surgical planning. The defect was repaired using a Prolene mesh, a synthetic material widely recognized in the literature for its efficacy in reinforcing the abdominal wall and reducing the risk of recurrence. The choice of mesh repair is particularly pertinent in the context of lumbar hernias, given the region’s complex anatomy and the significant mechanical forces exerted on the lumbar area, which necessitate a durable and robust repair to withstand physiological stress.

Postoperative management in patients who have undergone lumbar hernia repair is crucial for ensuring long-term success and minimizing complications. Close monitoring for potential issues such as infection, seroma formation, or mesh displacement is essential in the immediate postoperative period. In this case, the patient experienced an uneventful recovery, with no signs of complications such as infection or recurrence at follow-up. This positive outcome underscores the importance of meticulous surgical technique and vigilant postoperative care. Moreover, patient education plays a pivotal role in the recovery process, particularly in advising patients to modify their activities to avoid excessive strain on the repaired area. Patients are typically counseled on the importance of avoiding heavy lifting and engaging in gradual, supervised physical activity to promote healing and prevent recurrence. Complications associated with lumbar hernias may include incarceration or strangulation of the herniated tissue, where the protruded abdominal contents become trapped and compromise blood supply, leading to ischemia and potentially necrosis. This can cause severe pain, bowel obstruction, and systemic symptoms such as fever. Nerve compression from the hernia or associated edema can lead to radiculopathy, resulting in pain, numbness, or weakness in the lower limbs.

While lumbar hernias are rare, they should be considered in the differential diagnosis of patients presenting with unexplained lumbar swelling or discomfort, especially in those engaged in activities that increase intra-abdominal pressure. Advanced imaging techniques, particularly CT scans, are invaluable in the accurate diagnosis and preoperative assessment of these hernias, guiding effective surgical planning. The use of synthetic mesh in the surgical repair of lumbar hernias provides a reliable means of reinforcing the abdominal wall, thereby reducing the risk of recurrence. Early diagnosis and timely surgical intervention, coupled with careful postoperative management and patient education, are essential in achieving favorable outcomes and preventing complications in patients with lumbar hernias. This case exemplifies the need for awareness of this rare condition among clinicians and highlights the critical role of imaging and surgical expertise in its management.

## Conclusions

Lumbar hernias, though rare, should be considered in patients presenting with unexplained lumbar swelling, particularly when associated with activities that increase intra-abdominal pressure. Accurate diagnosis through imaging, especially CT scans, is crucial for effective management. Surgical repair, reinforced with mesh, offers a reliable solution with a low risk of recurrence. Early recognition and timely intervention are essential to prevent potential complications and ensure successful patient outcomes.
